# Genetic insights into the ‘sandwich fusion’ subtype of Klippel–Feil syndrome: novel FGFR2 mutations identified by 21 cases of whole-exome sequencing

**DOI:** 10.1186/s13023-024-03134-9

**Published:** 2024-04-01

**Authors:** Nanfang Xu, Kan-Lin Hung, Xiaoli Gong, Dongwei Fan, Yinglun Tian, Ming Yan, Yuan Wei, Shenglin Wang

**Affiliations:** 1 Department of Orthopaedics, Peking University Third Hospital, Haidian District, 49 North Garden Road, Beijing, 100191 China; 2 Engineering Research Center of Bone and Joint Precision Medicine, Beijing, China; 3 Beijing Key Laboratory of Spinal Disease Research, Beijing, China; 4 Department of Obstetrics and Gynecology, Peking University Third Hospital, Haidian District, 49 North Garden Road, Beijing, 100191 China

**Keywords:** Klippel–Feil syndrome, Atlantoaxial dislocation, Whole-exome sequencing, FGFR2

## Abstract

**Background:**

Klippel–Feil syndrome (KFS) is a rare congenital disorder characterized by the fusion of two or more cervical vertebrae during early prenatal development. This fusion results from a failure of segmentation during the first trimester. Although six genes have previously been associated with KFS, they account for only a small proportion of cases. Among the distinct subtypes of KFS, “sandwich fusion” involving concurrent fusion of C0-1 and C2-3 vertebrae is particularly noteworthy due to its heightened risk for atlantoaxial dislocation. In this study, we aimed to investigate novel candidate mutations in patients with “sandwich fusion.”

**Methods:**

We collected and analyzed clinical data from 21 patients diagnosed with “sandwich fusion.” Whole-exome sequencing (WES) was performed, followed by rigorous bioinformatics analyses. Our focus was on the six known KFS-related genes (*GDF3*, *GDF6*, *MEOX1*, *PAX1*, *RIPPLY2*, and *MYO18*). Suspicious mutations were subsequently validated through in vitro experiments.

**Results:**

Our investigation revealed two novel exonic mutations in the *FGFR2* gene, which had not previously been associated with KFS. Notably, the c.1750A > G variant in Exon 13 of *FGFR2* was situated within the tyrosine kinase domain of the protein, in close proximity to several established post-translational modification sites. In vitro experiments demonstrated that this certain mutation significantly impacted the function of FGFR2. Furthermore, we identified four heterozygous candidate variants in two genes (*PAX1* and *MYO18B*) in two patients, with three of these variants predicted to have potential clinical significance directly linked to KFS.

**Conclusions:**

This study encompassed the largest cohort of patients with the unique “sandwich fusion” subtype of KFS and employed WES to explore candidate mutations associated with this condition. Our findings unveiled novel variants in *PAX1*, *MYO18B*, and *FGFR2* as potential risk mutations specific to this subtype of KFS.

**Supplementary Information:**

The online version contains supplementary material available at 10.1186/s13023-024-03134-9.

## Introduction

Klippel–Feil syndrome (KFS) (OMIM 118100, 214300, 613702, 616549), initially delineated in 1912 by the French neurologists Maurice Klippel and Andre Feil, is characterized by a classic triad of clinical features, including a short neck, low posterior hairline, and restricted neck mobility. In contemporary parlance, KFS is defined as the congenital fusion of two or more cervical vertebrae, stemming from disrupted segmentation during the early stages of prenatal development [1]. Its reported incidence stands at 1 in 40,000 newborns, with a higher predilection for females [2, 3]. Beyond the classic triad, KFS presents a spectrum of clinical manifestations encompassing scoliosis, anomalies of the kidney and urinary tract, hearing impairment, congenital heart anomalies, and maxillofacial irregularities [4]. Remarkably, KFS exhibits considerable phenotypic heterogeneity, with fewer than half of afflicted individuals displaying the classic triad [5–10]. This diversity underscores the intricate pathophysiological processes and genetic underpinnings at play within this patient cohort. Indeed, KFS manifests various inheritance patterns [11], with previously identified candidate genes encompassing *GDF3* (OMIM 606522), *GDF6* (OMIM 601147), *MEOX1* (OMIM 600147), *PAX1* (OMIM 167411), *RIPPLY2* (OMIM 609891), and *MYO18* (OMIM 607295) [12].

In individuals with KFS, there is typically an increased segmental range of motion between the fused vertebral mass and the adjacent cranial and caudal cervical vertebrae. This compensatory mechanism aims to mitigate the limitations in neck mobility resulting from the fusion of multiple cervical segments. However, excessive compensation can precipitate instability at the cranial and caudal adjacent levels, fostering early degeneration due to overuse. This phenomenon is notably exemplified in patients with two fusion masses and only one intervening mobile segment, a configuration akin to a “sandwich fusion” scenario at the craniovertebral junction. These patients face a heightened risk of developing myelopathy stemming from spinal cord compression attributed to premature degeneration resulting from overuse. Given that the most frequently encountered level of fusion in KFS is C2-3 (74.1%) [13], our particular interest lies in cases where both C0-1 (occiput-atlas) and C2-3 are fused, with C1/2 representing the sole remaining mobile segment in the upper cervical spine. This distinctive KFS subtype was previously elucidated in our research as “sandwich fusion” of the cranial-vertebral junction [14]. Patients with “sandwich fusion” manifest an elevated propensity for atlantoaxial dislocation, frequently presenting at a younger age with more severe pathology necessitating surgical intervention compared to their non-“sandwich fusion” counterparts [15]. Moreover, the technical intricacies of their surgeries are amplified, often necessitating the versatile application of diverse internal fixation techniques. Hence, early detection and non-surgical management assume paramount significance in this patient cohort, underscoring the need for a comprehensive understanding of the pivotal pathogenic genes and pathways.

Our present study seeks to validate the six genes previously associated with KFS through genome-wide sequencing in the most extensive cohort of KFS patients exhibiting “sandwich fusion” at the cranial-vertebral junction. Additionally, our investigation aims to unearth novel candidate genes pertinent to this particularly high-risk population, providing a valuable link towards enhancing early detection and non-surgical therapeutic strategies.

## Methods

### Subjects

Ethical approval was granted by our hospital’s ethics committee, and informed consent was obtained from all participants. Surgical patients diagnosed with ‘sandwich fusion’ between February 2016 and October 2019 at a tertiary referral center for spine disorders were prospectively enrolled. Diagnosis of atlantoaxial instability or dislocation was established based on dynamic lateral X-rays and CT scans. Demographic data, clinical profiles, and neurological status assessed via the Japanese Orthopaedic Association (JOA) scale before and after surgery were documented. The JOA scale, comprising six domains (motor and sensory function in the upper and lower extremities, sensory function in the trunk, and bladder function), ranges from 0 to 4, 4, 2, 2, 2, and 3, respectively, with total scores ranging from 0 to 17.

### Preparation of DNA library

Blood samples from all patients were collected and forwarded to Beijing MyGenomics Inc., where genomic DNA (1–3 μg) was extracted from each specimen and fragmented to an average size of 180 bp using a Bioruptor sonicator (Diagenode, U.S.). Paired-end sequencing libraries were then prepared following standard protocols with NEBNext (Illumina, U.S.). This process included end repair, adapter ligation, and PCR enrichment.

### Targeted gene enrichment and sequencing

For each DNA pool, a targeted exome library with an insert size of 150–200 bp was constructed via an exome capture approach using the GenCap Custom Enrichment Kit (MyGenostics, China). Specifically, 1 μg of DNA library was mixed with GenCap gene panel probes (MyGenostics, China) in BL buffer. This mixture was heated to 95 °C for 7 min and then cooled to 65 °C for 2 min in a PCR machine. Subsequently, 23 μL of pre-warmed Buffer HY (MyGenostics, China) at 65 °C was added, and the mixture was maintained at 65 °C for 22 h for hybridization. Following hybridization, the sample underwent three rounds of washing with 50 μL MyOne magnetic beads (Life Technology, U.S.) in 500 μL of 1X binding buffer. The eluted sample was further processed with 80 μL of 1X binding buffer and 64 μL of 2X binding buffer, followed by rotation for 1 h with 80 μL MyOne beads (Life Technology, U.S.). The beads were subjected to three washes with WB1 buffer at room temperature for 15 min and three washes with WB3 buffer at 65 °C for 15 min. Finally, DNA was eluted with an Elution buffer and subjected to 15 cycles of amplification. Purification of the enriched libraries was performed using SPRI beads (Beckman Coulter, U.S.), followed by sequencing on a HiSeq X sequencer (Illumina, U.S.) for paired reads of 150 bp each.

### Quality control of sequencing data

Approximately 99.7% of base pairs aligned to the human reference genome (GRCh37, hg19), with 40.8% mapped to the targeted region. Quality control procedures included the capture of 5.6 Gb of exon regions and ensured that at least 96.1% of nucleotides were sequenced twenty times or more (Additional file 1: Table S1). These datasets offered high sensitivity and specificity for capturing coding mutations. On average, each sample yielded 13.1 Gb of sequence data in paired-end 2 × 150 bp reads.

### Bioinformatics analysis

Low-quality reads (< 80 bp) were excluded using Cutadapt (http://cutadapt.readthedocs.io/en/stable/). Clean reads were mapped to the human reference genome assembly GRCh37 (hg19) using BWA (http://bio-bwa.sourceforge.net/). Duplicate reads were eliminated with Picard (http://broadinstitute.github.io/picard/). Variations were detected in the mapping reads. For known gene validation, SNP and InDel variants were identified with GATK (https://software.broadinstitut.org/gatk/) Haplotype Caller, and GATK Variant Filtration was applied based on the following criteria: (a) mapping qualities < 30; (b) Total Mapping Quality Zero Reads < 4; (c) approximate read depth < 5; (d) QUAL < 50.0; (e) phred-scaled *p*-value (Fisher’s exact test) detecting strand bias > 10.0. Variant annotation was performed using ANNOVAR (http://annovar.openbioinformatics.org/en/latest/) with multiple databases, including 1000 Genome (http://www.1000genomes.org/), ESP6500 (http://evs.gs.washington.edu/EVS), dbSNP (http://www.ncbi.nlm.nih.gov/projects/SNP/), ExAC (http://exac.broadinstitute.org), HGMD (http://www.biobase-international.com/product/hgmd), ClinVar (http://www.ncbi.nlm.nih.gov/clinvar/), and the in-house MyGenostics. Pathogenic prediction employed SIFT (http://sift.jcvi.org), PolyPhen-2 (http://genetics.bwh.harvard.edu/pph2/), MutationTaster (http://www.mutationtaster.org/), and GERP++ (http://mendel.stanford.edu/SidowLab/downloads/gerp/index.html). For variants in coding regions, alignment of mutant transcripts across species was accomplished using CLUSTAL W (https://www.genome.jp/tools-bin/clustalw) and UGENE (http://ugene.net/). Functional alterations in non-coding regions were predicted with ESE Finder 3.0 (http://rulai.cshl.edu/tools/ESE) (Additional file 1: Fig. S1).

### Verification of gene mutations by Sanger sequencing

Pathogenic or likely pathogenic mutations identified by WES were confirmed through Sanger sequencing.

### In vitro experiment

The XhoI-wt-BamHI fragment was amplified using the synthetic FGFR2 coding sequence (CDS) as a template, and the primers pEGFP-C1-FGFR2-XhoI-F and pEGFP-C1-FGFR2-BamHI-R. Following double digestion with XhoI and BamHI, the pEGFP-C1-wt vector was ligated into the pEGFP-C1 vector.

The mut1-1 fragment was generated using the pEGFP-C1-wt vector as a template and the primers pEGFP-C1-FGFR2-XhoI-F and FGFR2-mut1-R in a PCR reaction. Similarly, the mut1-2 fragment was obtained using the primers FGFR2-mut1-F and pEGFP-C1-FGFR2-BamHI-R. Subsequently, mut1-1 and mut1-2 were mixed in a 1:1 ratio and used as templates for a second round of PCR amplification with the primers pEGFP-C1-FGFR2-XhoI-F and pEGFP-C1-FGFR2-BamHI-R. This process yielded the XhoI-mut1-BamHI fragment.

The pEGFP-C1-mut1 (c. 1213A > G) vector was obtained by double-digesting the mut1 fragment with XhoI and BamHI and then ligating it into the pEGFP-C1-wt vector. The pEGFP-C1-mut2 (c. 1750A > G) vector was similarly constructed.

Cultured 293 T cells were maintained in DMEM medium supplemented with 10% fetal bovine serum. The constructed wild-type and mutant eukaryotic recombinant expression vectors were transiently transfected into 293 T cells using Lipofectamine 2000 Transfection Reagent (Thermo Fisher Scientific, China). After 48 h of transfection, samples were collected for quantitative PCR (qPCR) analysis using Hieff ^®^qPCR SYBR Green Master Mix (YEASEN, Shanghai) and Western blot (WB) analysis.

## Results

### Clinical characteristics

**Fig. 1:**
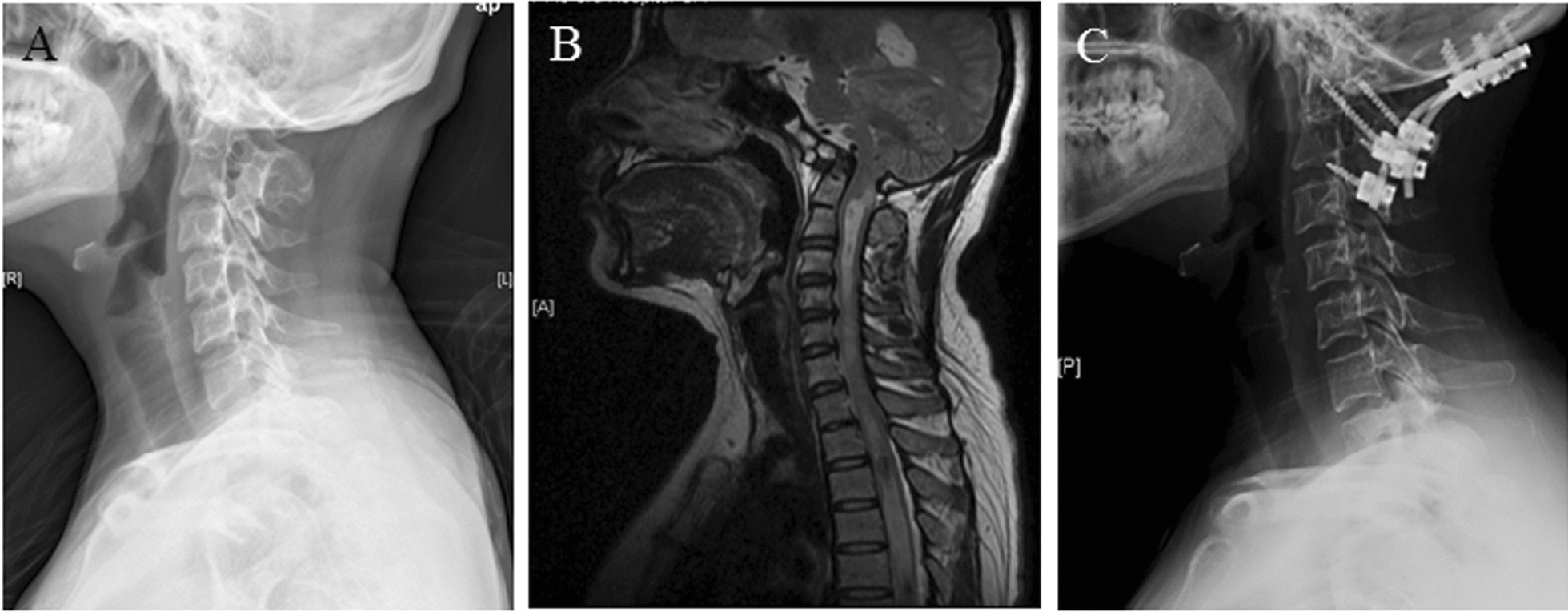
**A** Pre-operative lateral X-ray of the cervical spine demonstrating the classic “sandwich fusion” of both C0-1 and C2-3 and atlantoaxial dislocation. **B** Pre-operative sagittal reconstruction of MRI of the cervical spine demonstrating basilar invagination as well as anterior compression of the medulla by the cranially migrated odontoid tip. There was also significant syringomyelia. **C** Post-operative lateral X-ray of the cervical spine at the one-year follow-up demonstrating maintenance of reduction of atlantoaxial dislocation by occipito-cervical fixation consisting of six occipital screws, two C2 pedicle screws, and two C3 lateral mass screws

This study enrolled twenty-one patients with “sandwich fusion” who underwent surgical correction for atlantoaxial instability (66.7%) or dislocation (33.3%) (Fig. 1A). The cohort comprised 10 females (47.6%) and 11 males (52.4%), with an average age of 43 years (range 5–77). The mean age of disease onset was 36.5 years (range 1–65). Additional diagnoses included schizophrenia (Case 1), ossification of the posterior longitudinal ligament (Case 2), Sprengel’s deformity, and congenital fusion of thoracic vertebrae (Case 3), basilar invagination (Case 4), syringomyelia and diplopia (Case 15), torticollis (Case 19), and hearing loss (Case 21). Among the patients, 15 (71.4%) presented with myelopathic symptoms, and their JOA scores improved significantly following surgery, with an average increase of 1.6 points, exceeding the generally accepted minimum detectable change (MDC) or minimum clinically important difference (MCID) of JOA (Table 1).

**Table 1:** Demographics and clinical characteristics of the enrolled patients

No	Age	Gender	Ethnicity	Language	Origin	Age at disease manifestation	Diagnosis	Pre-op JOA	Post-op JOA	Hereditary
Case 1	40	F	Han	Chinese	China	27	AAI; schizophrenia	17	17	Sporadic
Case 2	56	F	Han	Chinese	China	56	AAI; ossificaiton of posterior longitudinal ligament	14	16	Sporadic
Case 3	11	M	Han	Chinese	China	1	AAD; Sprengel’s deformity; T3-6 congenital fusion	17	17	Sporadic
Case 4	64	M	Han	Chinese	China	64	AAD; basilar invagination	14	16	Sporadic
Case 5	58	F	Han	Chinese	China	58	AAI	16	16	Sporadic
Case 6	47	F	Han	Chinese	China	27	AAD	11	14	Sporadic
Case 7	41	F	Han	Chinese	China	41	AAI	17	17	Sporadic
Case 8	61	M	Han	Chinese	China	51	AAD	12	14	Sporadic
Case 9	39	M	Han	Chinese	China	39	AAD	15	16	Sporadic
Case 10	51	F	Han	Chinese	China	31	AAI	14	16	Sporadic
Case 11	50	F	Han	Chinese	China	50	AAI	14	15	Sporadic
Case 12	66	M	Han	Chinese	China	59	AAD	12	14	Sporadic
Case 13	55	M	Han	Chinese	China	45	AAD	11	14	Sporadic
Case 14	49	F	Han	Chinese	China	39	AAD	12	14	Sporadic
Case 15	28	F	Han	Chinese	China	28	AAD; syringomyelia; diplopia	14	15	Sporadic
Case 16	42	M	Han	Chinese	China	36	AAD	13	15	Sporadic
Case 17	36	M	Han	Chinese	China	36	AAD	16	16	Sporadic
Case 18	77	F	Han	Chinese	China	65	AAD	15	16	Sporadic
Case 19	11	M	Han	Chinese	China	5	AAD; torticollis	17	17	Sporadic
Case 20	5	M	Han	Chinese	China	1	AAI	17	17	Sporadic
Case 21	8	M	Han	Chinese	China	8	AAD; hearing loss	17	17	Sporadic

AAI, atlantoaxial instability, AAD, atlantoaxial dislocation

### Genetic analysis

Whole-exome sequencing (WES) was successfully conducted on all 21 patients, with a mean coverage depth of 100 × and at least 96.04% of bases covered by at least 20 reads. Four variants were identified as potentially clinically significant in association with KFS (Table 2).

**Table 2:** Variants identified by whole exome sequencing

No	Gene	Inheritance	Reference sequence	Nucleotide change	Amino-acid change	Genetic subregion	Heterogeneity	Chromosomal Loci	Mutation type	Reference
Case 7	MYO18B	AR	NM_032608	c.522C > T	p.D174D	EX4	Het	chr22:26164405	Likely pathogenic	-
	MYO18B	AR	NM_032608	c.1675G > A	p.E559K	EX6	Het	chr22:26166934	Likely pathogenic	-
Case 19	PAX1	AR	NM_006192	c.555G > A	p.K185K	EX2	Het	chr20:21687344	Uncertain	-
	PAX1	AR	NM_006192	c.1159C > T	p.L387F	EX4	Het	chr20:21689959	Likely pathogenic	-
Case 9	FGFR2	AD	NM_000141	c.1750A > G	p.M584V	EX13	Het	chr10:123256159	Likely pathogenic	-
Case 14	FGFR2	AD	NM_000141	c.1213A > G	p.K405E	EX9	Het	chr10:123274705	Likely pathogenic	-

AR, autosomal recessive; AD, autosomal dominant; EX, exon; Het, heterogeneous

**Fig. 2:**
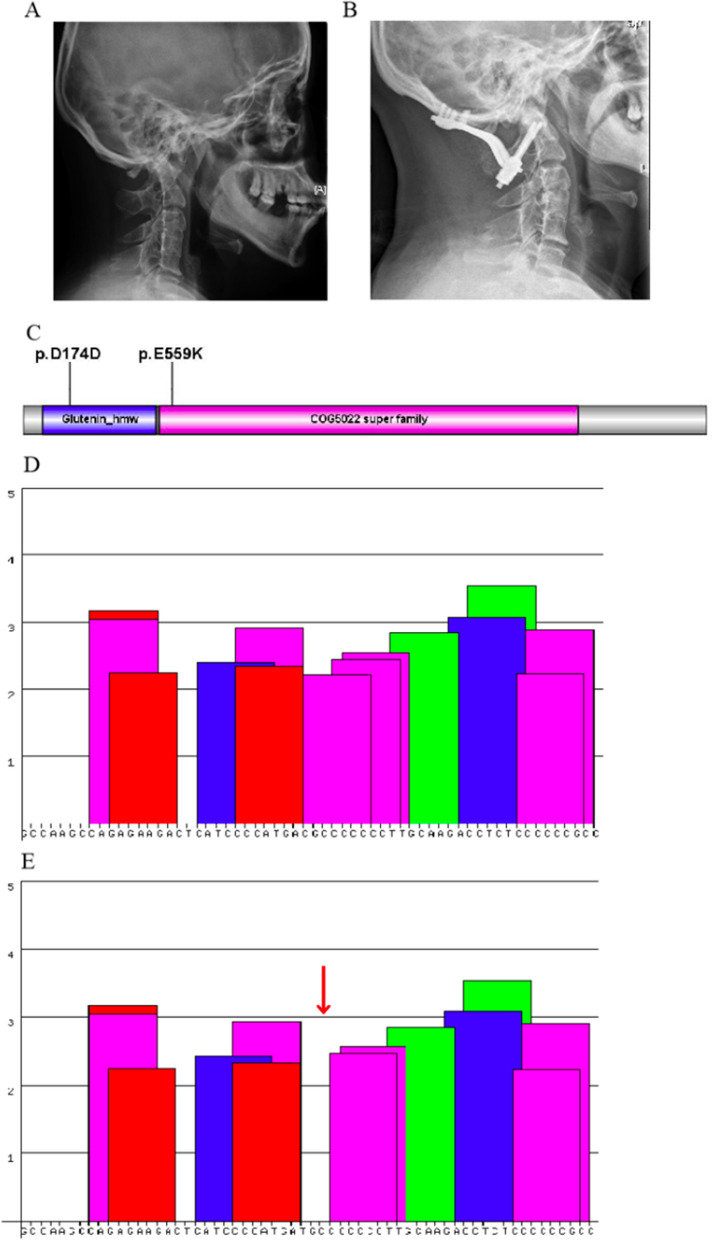
The analysis of variants c.522C > T (p.D174D) and c.1675G > A (p.E559K) in *MYO18B* in Patient 7. **A** Pre-operative lateral X-ray of the cervical spine demonstrating the classic “sandwich fusion” and atlantoaxial dislocation. **B** Post-operative lateral X-ray of the cervical spine at the one-year follow-up demonstrating maintenance of reduction of atlantoaxial dislocation by occipito-cervical fixation consisting of six occipital screws and two C2 pedicle screws. **C** Conserved Domain Search was performed, and the c.1675G > A (p.E559K) variant was located in the COG5022 superfamily domain of the protein, and the variant c.522C > T (p.D174D) was located in the Glutenin_hmw superfamily domain of the protein. **D**, **E** ESE finder3.0 was used to predict the effect of the mutations

In Case 7, variants c.522C > T (p.D174D) and c.1675G > A (p.E559K) were detected in myosin XVIIIB (*MYO18B*, OMIM 607295) (Fig. 2A, B). These *MYO18B* variants were rare in ExAC and the 1000 Genome databases but were not found in ESP6500, HGMD, or ClinVar. Both variants were considered compound heterozygous mutations. The c.1675G > A (p.E559K) variant was located within the COG5022 superfamily domain, while the c.522C > T (p.D174D) variant was found in the Glutenin_hmw superfamily domain (Fig. 2C). Per the 2019 American College of Medical Genetics and Genomics (ACMG) algorithm, both variants were classified as likely pathogenic. Additionally, the c.522C > T variant was predicted to affect splicing patterns by the splicing factor SRSF1, as supported by Human Splicing Finder analysis (Fig. 2D, E and Table 3).

**Table 3:** Prediction of c.522C > T in MYO18B by HSF (Human Splicing Finder)

Predicted signal	Prediction algorithm	cDNA Position	lnterpretation
New ESS Site	1—Sironi et al.—Motif 2		Creation of an exonic ESS site. Potential alteration of splicing
	ESR Sequences from Goren et al		
	3—Sironi et al.—Motif 3		

**Fig. 3:**
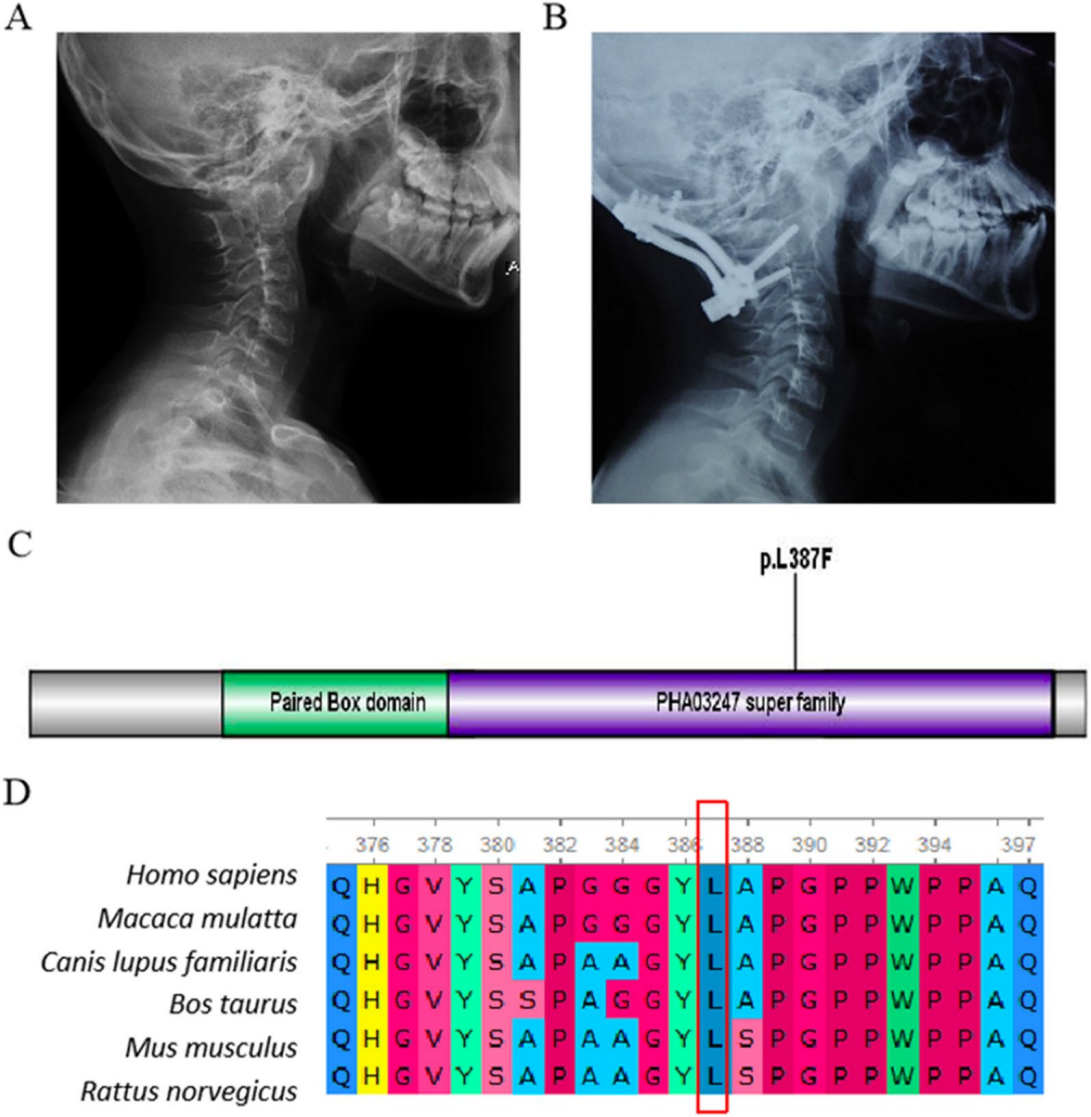
The analysis of variants c.555G > A (p.K185K) and c.1159C > T (p.L387F) in *PAX1* in Patient 19. **A** Pre-operative lateral X-ray of the cervical spine demonstrating the classic “sandwich fusion” and atlantoaxial dislocation. **B** Post-operative lateral X-ray of the cervical spine at the one-year follow-up demonstrating maintenance of reduction of atlantoaxial dislocation by occipito-cervical fixation consisting of six occipital screws, one C2 pedicle screw, and one C3 pedicle screw. **C** Conserved Domain Search was performed, and the variant c.1159C > T (p.L387F) was located in the PHA03247 superfamily domain of the protein. **D** Multiple sequence alignments revealed that the mutation site in PAX1 was highly evolutionarily conserved

In Case 19, variants c.555G > A (p.K185K) and c.1159C > T (p.L387F) were identified in paired box 1 (*PAX1*, OMIM 167411) (Fig. 3A, B). While the p.K185K variant was not rare, the p.L387F variant was novel and not present in ExAC, the 1000 Genome, or ESP6500 databases. The p.L387F variant resided in the PHA03247 superfamily domain of *PAX1* and was predicted to be deleterious by multiple tools (Fig. 3C). Multiple-sequence alignment confirmed the conservation of the p.L387F variant across species (Fig. 3D). The ACMG algorithm classified the c.1159C > T (p.L387F) variant in *PAX1* as likely pathogenic, and co-segregation analysis supported its inheritance pattern.

### Detection of novel risk gene

**Fig. 4:**
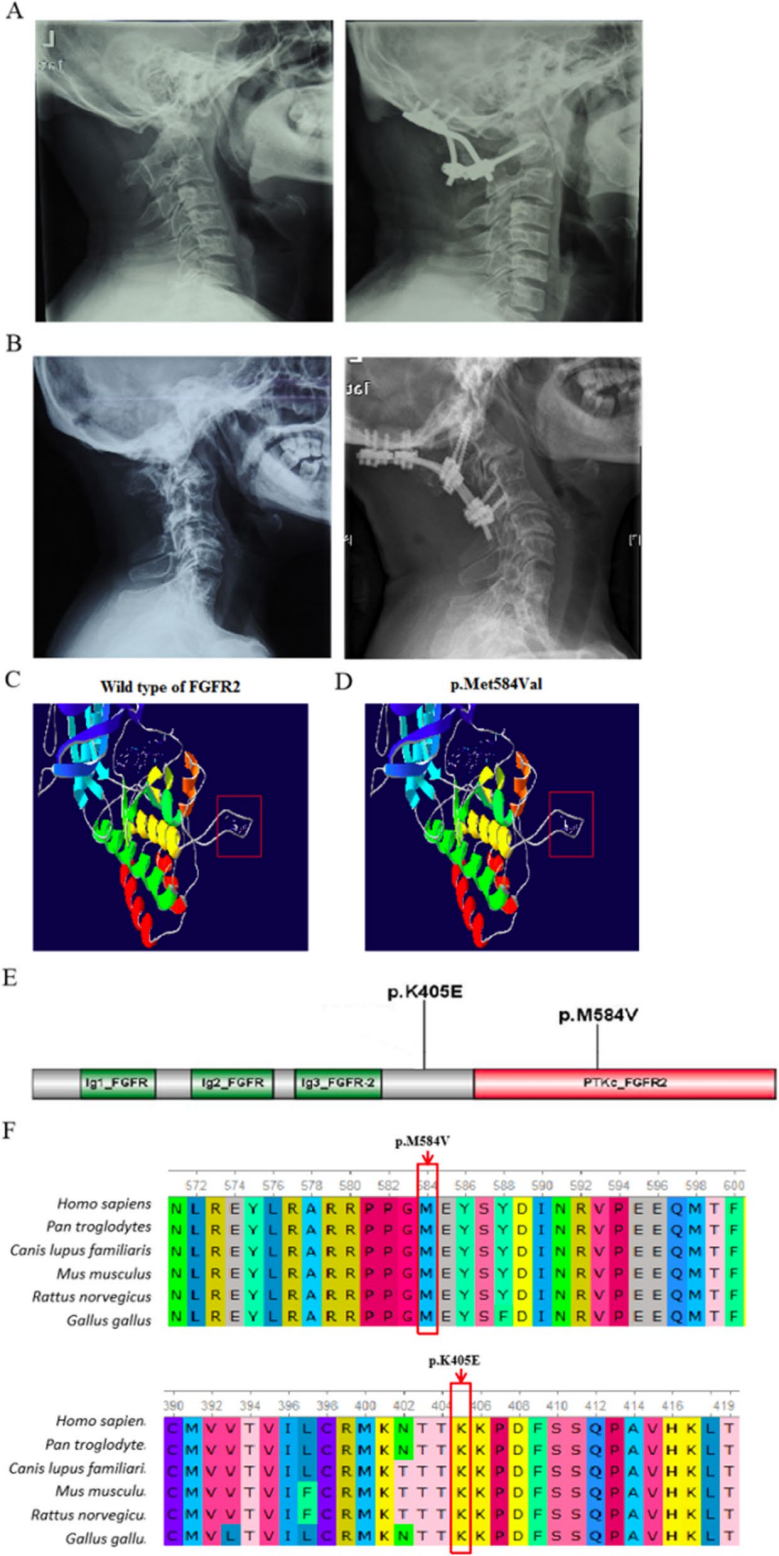
The analysis of variants c.1750A > G (p.M584V) and c.1213A > G (p.K405E) in *FGFR2* in Patients 14 and 9. **A**, **B** Pre- and Post-operative lateral X-ray of the cervical spine demonstrating the classic “sandwich deformity” with atlantoaxial dislocation in Patient 14 and Patient 9. **C**, **D** SWISSMODEL was used to predict the effects of the mutations in FGFR2 and significant differences between the structure of the mutated and wild-type were identified, indicating structural (red square) and functional change. **E** Conserved Domain Search was performed, and the variant c.1750A > G (p.M584V) was located in the PTKc domain of the protein. **F** Multiple sequence alignments revealed that the mutation sites were highly evolutionarily conserved

Two novel missense mutations, c.1750A > G (p.M584V) and c.1213A > G (p.K405E), were identified in *FGFR2* (OMIM 176943) through WES in Case 9 and 14, respectively (Fig. 4A, B) (Table 2). These mutations were not found in public databases, were classified as likely pathogenic by the ACMG algorithm, and were located in conserved regions across species (Fig. 4E, F). Functional prediction tools indicated the c.1750A > G variant as a damaging mutation (Table 4). The c.1750A > G (p.M584V) mutation was positioned within the catalytic domain of *FGFR2* and close to known post-translational modification sites [16–18].

**Table 4:** Prediction results of mutations found in FGFR2 according to different tools

No	Gene	Nucleotide change	SIFT	SIFT_Predict	PolyPhen_2	PolyPhen_2_Predict	MutationTaster	MutationTaster_Predict	GERP + +	GERP + + _Predict
Case 9	FGFR2	c.1213A > G	0.109	Tolerated	0.088	Benign	1	Disease_causing	5.28	Conserved
Case 14	FGFR2	c.1750A > G	0.041	Damaging	0.637	Possibly_damaging	1	Disease_causing	4.71	Conserved

In our in vitro experiments, both missense mutations had a substantial impact on mRNA and protein expression within *FGFR2*. The c.1213A > G (p.K405E) mutation resulted in a roughly 45% reduction in mRNA expression, while the c.1213A > G (p.M584V) mutation caused a striking 80% decrease (Data not shown). This significant reduction in mRNA expression underscores the potential deleterious effects of these mutations.

**Fig. 5:**
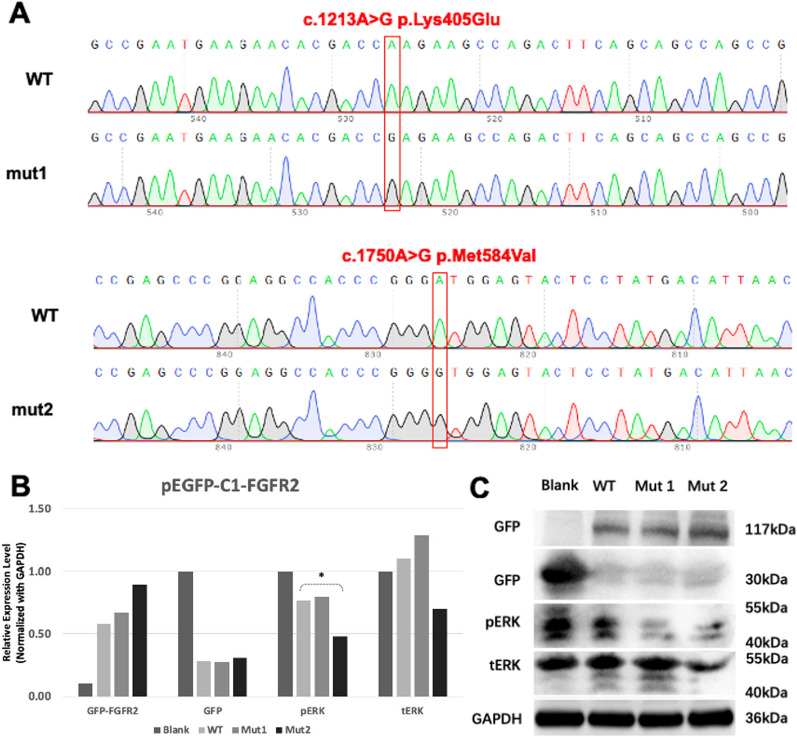
**A** Sequencing results showed that mutant mut1 (c.1213A > G (p.Lys405Glu)) and mut2 (c.1750A > G (p.Met584Val)) were successfully constructed. **B**, **C** The results of Western Blot showed that the pERK protein bands of mut2 decreased correspondingly, which stands for mut2 might lead to dysfunction of *FGFR2*. All the bands are normalized with GAPDH and Blank. *indicates *p* < 0.05

Moreover, our Western blot analysis unveiled a corresponding decrease in the phosphorylated ERK (pERK) protein bands specifically in the case of mut2, corresponding to the c.1213A > G (p.M584V) mutation (Fig. 5). Phosphorylated ERK is a well-established downstream effector of *FGFR2* signaling, known for its pivotal role in promoting bone differentiation. Consequently, this outcome strongly suggests that the particular mutation may indeed lead to the malfunction of *FGFR2*.

These findings shed light on the potential mechanisms through which *FGFR2* mutations can disrupt normal cellular processes and contribute to the development of conditions like the “sandwich fusion” subtype of Klippel–Feil Syndrome (KFS). Further investigations are warranted to elucidate the precise pathways affected by these mutations and their implications for bone development and fusion at the craniovertebral junction.

## Discussion

The pathogenesis of Klippel–Feil Syndrome (KFS) is often attributed to vertebral segmentation failure in the cervical region, resulting from defective somitogenesis [19]. Previous investigations have unveiled both autosomal dominant and autosomal recessive inheritance patterns in KFS patients. Six genes (*MYO18B*, OMIM 607295; *MEOX1*, OMIM 600147; *GDF6*, OMIM 601147; *GDF3*, OMIM 606522; *PAX1*, OMIM 167411; and *RIPPLY2*, OMIM 609891) have been implicated as pathogenic culprits. However, the genetic basis of this rare condition remains largely enigmatic due to its phenotypical heterogeneity, and only a fraction of KFS cases can be adequately explained by these six genes [20].

In our study, we endeavored to validate the involvement of these genes within our extensive KFS cohort, one of the largest among KFS patients in existing literature. Our scrutiny uncovered four heterozygous candidate variants within two genes, *PAX1* and *MYO18B*, in two patients (Table 2). *PAX1*, situated on chromosome 20p11, encodes a member of the paired-box (PAX) family of transcription factors. These factors are pivotal during fetal development, playing a central role in vertebral column development. *MYO18B*, located on chromosome 22q12, encodes myosin XVIIIB, a protein functioning as a homodimer. It is believed to regulate muscle-specific genes in the nucleus and influence intracellular trafficking in the cytoplasm by interacting with F-actin. Of the four novel variants we identified (including both *MYO18B* variants and the c.1159C > T variant of *PAX1*), three were predicted to have potential clinical significance directly linked to KFS, although further confirmation of their significance is warranted.

A noteworthy link emerges when examining the literature: KFS was initially described in association with craniosynostosis in the 1990s, with subsequent authors corroborating these findings [21–25]. This suggests a potential high level of relatedness, both phenotypically and at the molecular level, between these two conditions. In 2008, Tassabehji et al. reported that KFS caused by a loss-of-function mutation of *GDF6* was associated with craniofacial malformation [26]. Craniosynostosis is commonly linked to mutations in FGFRs, with *FGFR2* (OMIM 176943) being the most frequently implicated in craniosynostosis syndromes. During early embryonic development, intricate differentiation processes take place at the craniovertebral junction, involving the differentiation of neural crest cells through *Twist1/2*-regulated FGF and BMP signaling pathways. The perturbation of both BMP signaling pathways, affected by *GDF6* mutations, and FGF signaling pathways, affected by *FGFR2* mutations, may collectively contribute to the complex phenotype observed in patients with cervical segmental insufficiency and craniosynostosis.

Our study yielded two novel exonic mutations (c.1750A > G and c.1213A > G) within *FGFR2*, genes not previously associated with KFS. *FGFR2* serves as a receptor for fibroblast growth factors (FGF), with highly conserved amino acid sequences across evolution [26]. The extracellular portion of *FGFR2* interacts with fibroblast growth factors, thereby stimulating downstream signaling pathways critical for cellular mitogenesis and differentiation [27]. In contrast, the intracellular portion of *FGFR2* houses the classic catalytic region of tyrosine kinase. Mutations in *FGFR2* have been linked to numerous medical syndromes, such as Apert syndrome (OMIM 101200) [28], Beare-Stevenson cutis gyrata syndrome (OMIM 123790) [29], Crouzon syndrome (OMIM 123500) [28], Jackson-Weiss syndrome (OMIM 123150) [29], Lacrimo-Auriculo-Dento-Digital (LADD) syndrome (OMIM 149730) [30], and Pfeiffer syndrome (OMIM 101600) [29]. These syndromes often share a common developmental bony abnormality characterized by craniosynostosis—a premature fusion of cranial sutures that leads to cranial deformation and distinctive facial features.

Furthermore, recent reviews on *FGFR2*-related craniosynostosis syndromes have identified additional skeletal anomalies such as syndactyly, carpal and tarsal fusion, as well as synostosis of the radius and humerus among patients with Apert syndrome (OMIM 101200), Jackson-Weiss syndrome (OMIM 123150), and Pfeiffer syndrome (OMIM 101600), respectively [31]. Notably, a study on seven patients with Apert syndrome (OMIM 101200) reported multiple cervical vertebrae fusion [29], and a three-generation family with Crouzon syndrome (OMIM 123500) presented with cervical spine deformities [32].

*FGFR2* exhibits high expression in differentiating osteoblasts and osteoprogenitors. Downstream pathways regulated by *FGF/FGFR2* signaling play a pivotal role in the proliferation, differentiation, and apoptosis of osteoprogenitors [33]. Comprising more than 20 exons, *FGFR2* has been scrutinized for mutations, with Exons 9 and 10 encoding the extracellular immunoglobulin-like III (IgIII) domain—a hotspot for mutations in craniosynostosis syndromes [34]. Mutations have also been observed in Exons 13, 14, 15, 16, and 17, collectively encoding the tyrosine kinase region of the FGF receptor [35]. The novel variants uncovered in our study, namely c.1213A > G from Exon 9 and c.1750A > G from Exon 13, were not previously reported in patients with craniosynostosis syndromes. Nevertheless, their proximity to known pathogenic mutations suggests a plausible underlying molecular mechanism linking “sandwich fusion” and craniosynostosis. This connection may provide an explanation for the apparent phenotypic resemblance, where fusion occurs either at the craniovertebral junction or within cranial bones.

Our research provides robust evidence indicating that the identified *FGFR2* c.1750A > G variants exert a profound negative influence on protein expression. This substantial reduction in protein levels likely exacerbates functional impairment, thereby contributing significantly to the development of the distinctive “sandwich deformity” observed in the “sandwich fusion” subtype of Klippel–Feil Syndrome (KFS).

Notably, the M584V mutation appears to introduce a steric hindrance effect within the protein’s structural region. This structural alteration disrupts the proper folding of *FGFR2*, potentially compromising its catalytic capability and normal cellular function (Additional file 1: Fig. S2).

Of paramount importance is the insight gained from prior research regarding the role of FGFRs in somitogenesis, particularly their interaction with EphA4, a receptor tyrosine kinase. This intricate interplay involves bidirectional cross-phosphorylation and plays a pivotal role in the formation of somite boundaries—a process critical for facilitating the transition of mesenchymal cells to epithelial cells [36].

These findings emphasize the intricate molecular mechanisms underlying the development of KFS, especially within the “sandwich fusion” subtype. It is imperative that future research delves further into the precise pathways influenced by *FGFR2* mutations and their direct impact on the development and fusion of the craniovertebral junction. In light of these observations, we strongly advocate for comprehensive investigations to unravel the specific implications of *FGFR2* mutations. Such studies have the potential to provide invaluable insights into their association with the pathogenesis of the “sandwich fusion” subtype of KFS.

The limitations of this study encompass a small sample size (21 cases), dependence on bioinformatics predictions, and the absence of animal model validation. Prior KFS gene studies focused on West Asia-Mediterranean pedigrees, whereas this study only included the Han population, lacking detailed demographic comparison. Additionally, KFS patients often present with multisystem malformations, necessitating multidisciplinary diagnosis and treatment. Isolated studies may introduce clinical data bias, impeding a comprehensive understanding of KFS’s unique characteristics. This research endeavor will bridge the existing knowledge gap, advancing our understanding of the shared molecular mechanisms between these conditions and potentially opening new avenues for therapeutic interventions.

## Conclusions

In conclusion, our current whole-exome sequencing (WES) study conducted on a distinct subtype of Klippel–Feil syndrome (KFS) patients has shed light on two novel variants within the *FGFR2* gene, a gene not previously implicated in KFS pathogenesis. Furthermore, we have identified four novel mutations in genes associated with KFS according to existing literature. These findings unveil potential new genetic loci contributing to the pathogenesis of the “sandwich fusion” subtype of KFS.

### Supplementary Information

**Additional file 1: Fig. S1.** Bioinformatics analysis workflow (the filtration strategy). Samples were initially mapped to the human reference genome GRCh37 (hg19) using BWA, followed by the removal of low-quality reads (< 80 bps) using CutAdapt and elimination of duplicate reads with Picard. Subsequently, SNP and INDEL variants were detected using GATK. In the third step, various software tools, including ANNOVAR, 1000Genome, ESP6500, dbSNP, ExAC, HGMD, ClinVar, were employed for sample annotation and further exclusion of deep intron variants. Comparative analysis with databases such as 1000Genome, ESP6500, and ExAC was performed to eliminate common SNPs. Pathogenicity prediction scores were determined using SIFT, Polyphen-2, MutationTaster, and Gerp++. GLUSTAL W and UGENE were utilized for cross-species comparison to ascertain sequence conservation, while ESE Finder 3.0 was employed to predict potential changes in protein structure. Finally, all suspicious variants underwent Sanger sequencing for further validation. **Fig. S2.** The animated diagram of *FGFR2* (RCSB PDB number: 3B2T) and the arrowhead indicated the M584 site. **A** Ball-and-stick model of *FGFR2*; the sequence from 582 to 596 is shown in a dotted line. **B** Molecular surface model of *FGFR2*; the pink bulky region is P582. Mutation of M584V might result in a steric hindrance effect in the structural region of the protein and affect the folding of *FGFR2* and reduce its catalytic capability. **Table S1.** Quality control of sequencing data.

## Data Availability

Data and materials are available upon reasonable request to the corresponding authors. All data were uploaded to the Sequence Read Archive of NIH (SRA accession numbers: SAMN39948731-50) (url: https://dataview.ncbi.nlm.nih.gov/object/SAMN39948731-50).
