# Supplementary material for: Genetic insights into the ‘sandwich fusion’ subtype of Klippel–Feil syndrome: novel FGFR2 mutations identified by 21 cases of whole-exome sequencing
Source: Orphanet J Rare Dis. 2024 Apr 1;19:141. doi: 10.1186/s13023-024-03134-9 (PMC10985996; doi:10.1186/s13023-024-03134-9)
Supplement: Supplementary file 1 — Additional file 1: Fig. S1. Bioinformatics analysis workflow (the filtration strategy). Samples were initially mapped to the human reference genome GRCh37 (hg19) using BWA, followed by the removal of low-quality reads (< 80 bps) using CutAdapt and elimination of duplicate reads with Picard. Subsequently, SNP and INDEL variants were detected using GATK. In the third step, various software tools, including ANNOVAR, 1000Genome, ESP6500, dbSNP, ExAC, HGMD, ClinVar, were employed for sample annotation and further exclusion of deep intron variants. Comparative analysis with databases such as 1000Genome, ESP6500, and ExAC was performed to eliminate common SNPs. Pathogenicity prediction scores were determined using SIFT, Polyphen-2, MutationTaster, and Gerp++. GLUSTAL W and UGENE were utilized for cross-species comparison to ascertain sequence conservation, while ESE Finder 3.0 was employed to predict potential changes in protein structure. Finally, all suspicious variants underwent Sanger sequencing for further validation. Fig. S2. The animated diagram of FGFR2 (RCSB PDB number: 3B2T) and the arrowhead indicated the M584 site. A Ball-and-stick model of FGFR2; the sequence from 582 to 596 is shown in a dotted line. B Molecular surface model of FGFR2; the pink bulky region is P582. Mutation of M584V might result in a steric hindrance effect in the structural region of the protein and affect the folding of FGFR2 and reduce its catalytic capability. Table S1. Quality control of sequencing data. [file 13023_2024_3134_MOESM1_ESM.docx]

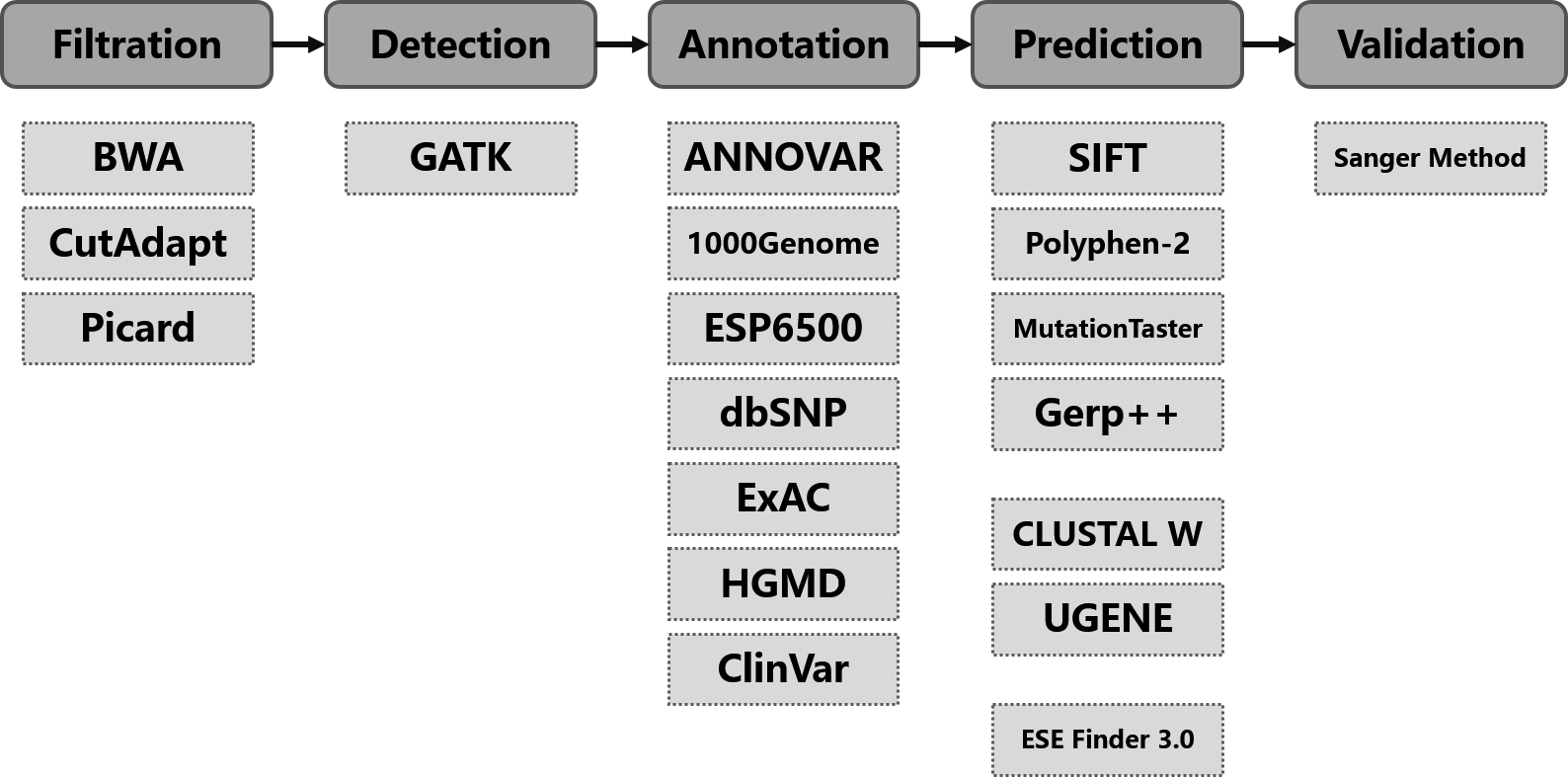


**Figure S1.** Bioinformatics analysis workflow (the filtration strategy). Samples were initially mapped to the human reference genome GRCh37 (hg19) using BWA, followed by the removal of low-quality reads (<80 bps) using CutAdapt and elimination of duplicate reads with Picard. Subsequently, SNP and INDEL variants were detected using GATK. In the third step, various software tools, including ANNOVAR, 1000Genome, ESP6500, dbSNP, ExAC, HGMD, ClinVar, were employed for sample annotation and further exclusion of deep intron variants. Comparative analysis with databases such as 1000Genome, ESP6500, and ExAC was performed to eliminate common SNPs. Pathogenicity prediction scores were determined using SIFT, Polyphen-2, MutationTaster, and Gerp++. GLUSTAL W and UGENE were utilized for cross-species comparison to ascertain sequence conservation, while ESE Finder 3.0 was employed to predict potential changes in protein structure. Finally, all suspicious variants underwent Sanger sequencing for further validation.


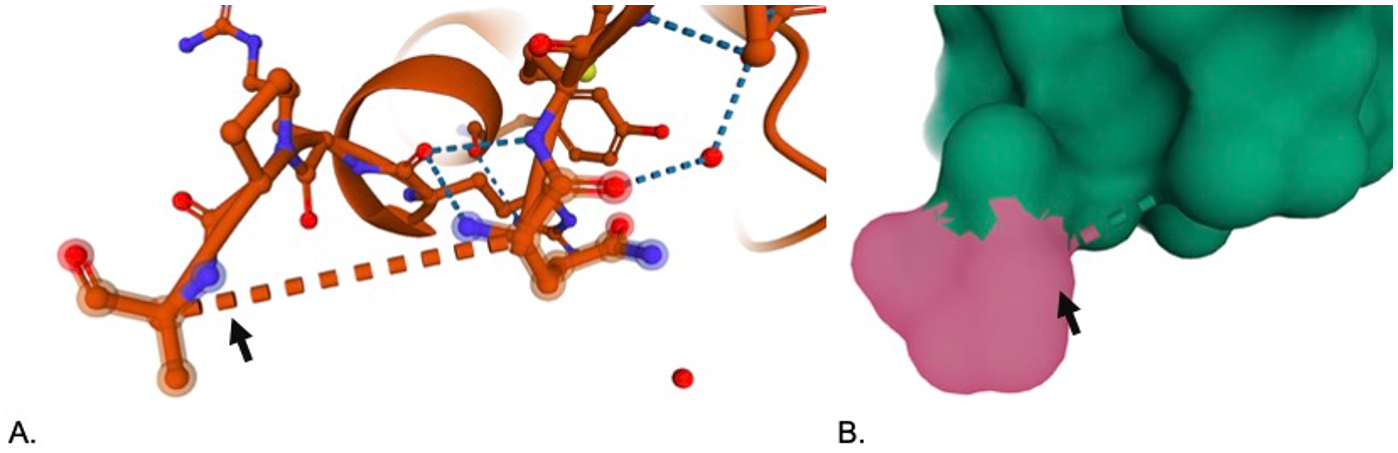


**Figure S2.** The animated diagram of FGFR2 (RCSB PDB number: 3B2T) and the arrow head indicated the M584 site. A. Ball-and-stick model of FGFR2; the sequence from 582 to 596 is shown in dotted line. B. Molecular surface model of FGFR2; the pink bulky region is P582. Mutation of M584V might result in a steric hindrance effect in the structural region of the protein and affect the folding of FGFR2 and reduce its catalytic capability.

Table S1. Quality control of sequencing data.

| No. | Raw_data(Gb) | Aligned(Gb) | Aligned | Effective bases on target (Gb) | Fraction of effective bases on target | Fraction of target covered with at least 20X |
| --- | --- | --- | --- | --- | --- | --- |
|  |  |  |  |  |  |  |
| Case 1 | 13.32 | 13.08 | 99.74% | 4.95 | 37.82% | 95.22% |
| Case 2 | 12.39 | 12.16 | 99.63% | 5.02 | 41.31% | 96.08% |
| Case 3 | 10.74 | 10.56 | 99.75% | 4.19 | 39.69% | 93.32% |
| Case 4 | 12.59 | 12.4 | 99.81% | 5.49 | 44.26% | 96.52% |
| Case 5 | 11.08 | 10.9 | 99.72% | 4.5 | 41.30% | 94.70% |
| Case 6 | 19.48 | 19.15 | 99.72% | 7.68 | 40.10% | 98.03% |
| Case 7 | 16.03 | 15.76 | 99.74% | 6.27 | 39.81% | 97.02% |
| Case 8 | 14.41 | 14.18 | 99.75% | 5.57 | 39.27% | 96.46% |
| Case 9 | 15.11 | 14.86 | 99.73% | 5.94 | 40.00% | 97.17% |
| Case 10 | 14.63 | 14.41 | 99.79% | 6.18 | 42.90% | 96.99% |
| Case 11 | 10.98 | 10.8 | 99.76% | 4.45 | 41.22% | 94.77% |
| Case 12 | 11.16 | 10.99 | 99.74% | 4.56 | 41.53% | 94.11% |
| Case 13 | 14.45 | 14.22 | 99.77% | 5.53 | 38.90% | 96.72% |
| Case 14 | 12.1 | 11.88 | 99.69% | 4.61 | 38.84% | 95.10% |
| Case 15 | 14.67 | 14.46 | 99.80% | 6.07 | 41.96% | 96.69% |
| Case 16 | 11.06 | 10.88 | 99.75% | 4.24 | 39.03% | 93.75% |
| Case 17 | 14.04 | 13.81 | 99.74% | 5.47 | 39.63% | 96.68% |
| Case 18 | 15.41 | 15.18 | 99.76% | 6.27 | 41.33% | 96.69% |
| Case 19 | 16.25 | 16.02 | 99.81% | 6.72 | 41.92% | 97.88% |
| Case 20 | 12.56 | 12.34 | 99.73% | 5.16 | 41.80% | 95.78% |
| Case 21 | 14.81 | 14.62 | 99.75% | 6.13 | 41.91% | 97.06% |

Table S2. Primers used in experiments.

|  | Primer Name | Primer Sequence |
| --- | --- | --- |
| Vector Construction | pEGFP-C1-FGFR2-XhoI-F | AGATCTCGAGctATGGTCAGCTGGGGTCGTTTC |
|  | pEGFP-C1-FGFR2-BamHI-R | CGGTGGATCCTCATGTTTTAACACTGCCGTT |
|  | FGFR2-mut1-F | ATGAAGAACACGACCGAGAAGCCAGACTTCA |
|  | FGFR2-mut1-R | TGAAGTCTGGCTTCTCGGTCGTGTTCTTCAT |
|  | FGFR2-mut2-F | CGGAGGCCACCCGGGGTGGAGTACTCCTATG |
|  | FGFR2-mut2-R | CATAGGAGTACTCCACCCCGGGTGGCCTCCG |
| qPCR | FGFR2-GFP-qPCR-F | CAAAGACCCCAACGAGAAGC |
|  | FGFR2-GFP-qPCR-R | GGTGGCTCTTCTGGCTCTAA |
